# [1,1′-Dibenzyl-2,2′-(2-oxapropane-1,3-diyl)di(1*H*-benzimidazole)-κ^3^ *N* ^3^,*O*,*N* ^3′^]bis(2,4,6-trinitrophenolato-κ*O* ^1^)manganese(II)

**DOI:** 10.1107/S1600536812026037

**Published:** 2012-06-16

**Authors:** Congfen Li, Fan Kou, Xinghan Li, Hao Wu, Huilu Wu

**Affiliations:** a School of Chemical and Biological Engineering, Lanzhou Jiaotong University, Lanzhou 730070, P.R. China

## Abstract

In the title complex, [Mn(C_6_H_2_N_3_O_7_)_2_(C_30_H_26_N_4_O)], the Mn^II^ atom is coordinated by the tridentate bis-benzimidazole ligand and two atoms of the picrate anions, in a distorted square-pyramidal geometry (τ = 0.038). One nitro O atom of one picrate ion is disordered over two sites with occupancies of 0.54 (5) and 0.46 (5).

## Related literature

For related structures, see: Addison *et al.* (1983 ▶); Wu *et al.* (2009 ▶, 2011 ▶). For the computation of the τ parameter describing the distortion of a square-pyramidal geometry, see: Addison *et al.* (1984 ▶).
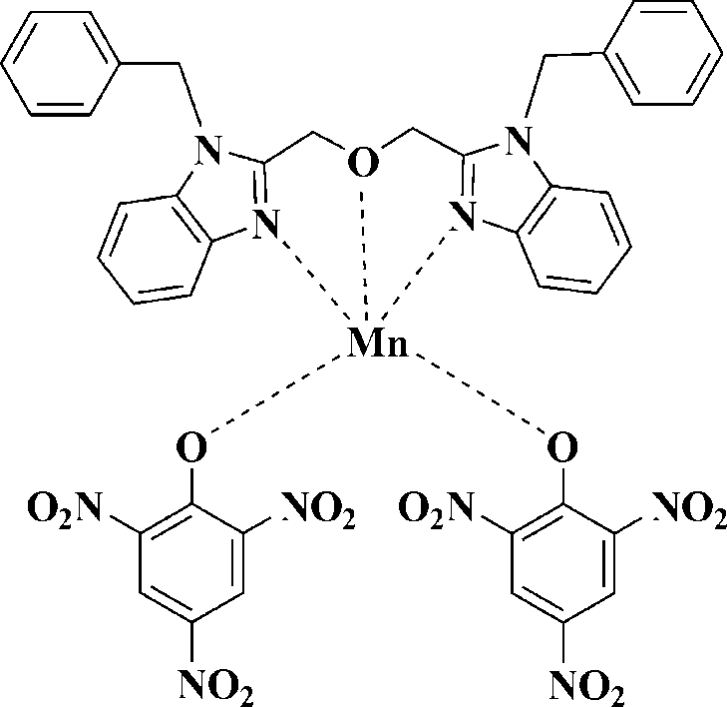


## Experimental

### 

#### Crystal data

[Mn(C_6_H_2_N_3_O_7_)_2_(C_30_H_26_N_4_O)]
*M* *_r_* = 969.70
Orthorhombic, 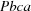

*a* = 22.323 (6) Å
*b* = 10.937 (3) Å
*c* = 34.130 (9) Å
*V* = 8333 (4) Å^3^
*Z* = 8
Mo *K*α radiation
μ = 0.40 mm^−1^
*T* = 293 K
0.28 × 0.21 × 0.15 mm

#### Data collection

Bruker APEXII CCD diffractometer
Absorption correction: multi-scan (*SADABS*; Sheldrick, 1996 ▶) *T*_min_ = 0.895, *T*_max_ = 0.942
54714 measured reflections
10276 independent reflections
5820 reflections with *I* > 2σ(*I*)
*R*_int_ = 0.060

#### Refinement

*R*[*F* ^2^ > 2σ(*F* ^2^)] = 0.058
*wR*(*F* ^2^) = 0.136
*S* = 1.01
10276 reflections
623 parameters
1 restraint
H-atom parameters constrained
Δρ_max_ = 0.53 e Å^−3^
Δρ_min_ = −0.42 e Å^−3^

### 

Data collection: *APEX2* (Bruker, 2007 ▶); cell refinement: *SAINT* (Bruker, 2007 ▶); data reduction: *SAINT*; program(s) used to solve structure: *SHELXS97* (Sheldrick, 2008 ▶); program(s) used to refine structure: *SHELXL97* (Sheldrick, 2008 ▶); molecular graphics: *SHELXTL* (Sheldrick, 2008 ▶); software used to prepare material for publication: *SHELXTL*.

## Supplementary Material

Crystal structure: contains datablock(s) global, I. DOI: 10.1107/S1600536812026037/bh2434sup1.cif

Structure factors: contains datablock(s) I. DOI: 10.1107/S1600536812026037/bh2434Isup2.hkl

Additional supplementary materials:  crystallographic information; 3D view; checkCIF report

## Figures and Tables

**Fig. 1.**
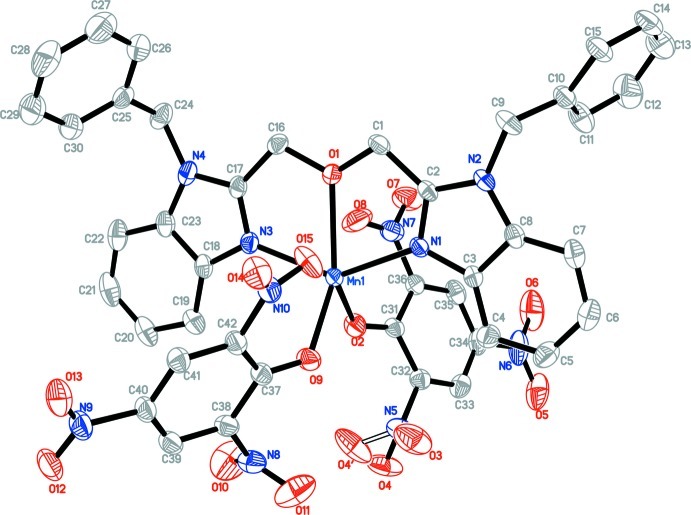
The molecular structure of the title compound. Displacement ellipsoids are drawn at the 30% probability level and H atoms have been omitted for clarity.

## supplementary crystallographic information

### Comment

Interest in bis(2-benzimidazolyl)alkanes and their derivatives is widespread (Addison *et al.*, 1983). We have previously reported the crystal structures of some related complexes (Wu *et al.*, 2009, 2011), and now complete this work with a new Mn(II) complex.

The complex crystallizes in the *Pbca* space group and its structure is shown in Fig. 1. The Mn(II) ion is coordinated by one tridentate 1,3-bis(1-benzylbenzimidazol-2-yl)-2-oxopropane ligand and two picrate ions, in a five-coordinated distorted square-pyramidal geometry. The distortion in the coordination polyhedron (τ) from a perfect trigonal bipyramidal geometry (τ = 1) toward a regular tetragonal pyramid (τ = 0) has been calculated according to the usual method (Addison *et al.*, 1984): τ = (β-α/60) where β and α are the largest bong angles around the metal center. In the title complex, O1, N1, O2, and N3 make up the basal plane, where the largest deviation for the four atoms is 0.384 Å, while the Mn atom is shifted by 0.585 Å from the mean plane. The apical site is occupied by O9, and τ = 0.038, since β = O9—Mn1—O1 and α = N3—Mn1—N1. One O atom is disordered over two sites (O4 and O4') in a picrate ligand, and the occupancies were refined, converging to 0.54 (5) and 0.46 (5).

### Experimental

To a stirred solution of 1,3-bis(1-benzylbenzimidazol-2-yl)-2-oxopropane (0.1832 g, 0.40 mmol) in hot MeOH (5 ml) was added Mn(II) picrate (0.1032 g, 0.20 mmol) in MeOH (5 ml). A deep yellow crystalline product formed rapidly. The precipitate was filtered off, washed with MeOH, and dried *in vacuo*. The dried precipitate was dissolved in DMF, resulting in a brown solution. Brown crystals suitable for X-ray diffraction studies were obtained by ether diffusion into DMF, after several days, at room temperature.

### Refinement

All H atoms were visible in a difference electron map, but were placed in idealized positions and refined in a riding-model approximation with C—H distances ranging from 0.93 to 0.97 Å and with *U*_iso_(H) = 1.2 *U*_eq_(carrier C). One picrate O atom is disordered over two sites, O4 and O4', with refined occupancies of 0.54 (5) and 0.46 (5).

### Crystal data

**Table:**

[Mn(C_6_H_2_N_3_O_7_)_2_(C_30_H_26_N_4_O)]	*F*(000) = 3976
*M**_r_* = 969.70	*D*_x_ = 1.546 Mg m^−^^3^
Orthorhombic, *P**b**c**a*	Mo *K*α radiation, λ = 0.71073 Å
Hall symbol: -P 2ac 2ab	Cell parameters from 7261 reflections
*a* = 22.323 (6) Å	θ = 2.4–22.5°
*b* = 10.937 (3) Å	µ = 0.40 mm^−^^1^
*c* = 34.130 (9) Å	*T* = 293 K
*V* = 8333 (4) Å^3^	Block, brown
*Z* = 8	0.28 × 0.21 × 0.15 mm

### Data collection

**Table:**

Bruker APEXII CCD diffractometer	10276 independent reflections
Radiation source: fine-focus sealed tube	5820 reflections with *I* > 2σ(*I*)
Graphite monochromator	*R*_int_ = 0.060
ω scans	θ_max_ = 28.4°, θ_min_ = 2.2°
Absorption correction: multi-scan (*SADABS*; Sheldrick, 1996)	*h* = −18→29
*T*_min_ = 0.895, *T*_max_ = 0.942	*k* = −14→13
54714 measured reflections	*l* = −45→45

### Refinement

**Table:**

Refinement on *F*^2^	Primary atom site location: structure-invariant direct methods
Least-squares matrix: full	Secondary atom site location: difference Fourier map
*R*[*F*^2^ > 2σ(*F*^2^)] = 0.058	Hydrogen site location: inferred from neighbouring sites
*wR*(*F*^2^) = 0.136	H-atom parameters constrained
*S* = 1.01	*w* = 1/[σ^2^(*F*_o_^2^) + (0.0392*P*)^2^ + 7.9255*P*] where *P* = (*F*_o_^2^ + 2*F*_c_^2^)/3
10276 reflections	(Δ/σ)_max_ = 0.002
623 parameters	Δρ_max_ = 0.53 e Å^−^^3^
1 restraint	Δρ_min_ = −0.42 e Å^−^^3^
0 constraints	

### Fractional atomic coordinates and isotropic or equivalent isotropic displacement parameters (Å^2^)

**Table:**

	*x*	*y*	*z*	*U*_iso_*/*U*_eq_	Occ. (<1)
C1	1.01293 (13)	0.4186 (2)	0.10980 (9)	0.0408 (7)	
H1A	0.9774	0.3757	0.1009	0.049*	
H1B	1.0422	0.3590	0.1186	0.049*	
C2	1.03816 (12)	0.4960 (2)	0.07773 (8)	0.0351 (6)	
C3	1.06411 (12)	0.6554 (3)	0.04428 (8)	0.0366 (6)	
C4	1.07450 (14)	0.7728 (3)	0.03043 (9)	0.0496 (8)	
H4	1.0669	0.8414	0.0458	0.059*	
C5	1.09660 (16)	0.7831 (3)	−0.00716 (10)	0.0616 (9)	
H5A	1.1041	0.8604	−0.0174	0.074*	
C6	1.10795 (16)	0.6806 (4)	−0.03014 (10)	0.0627 (10)	
H6	1.1226	0.6912	−0.0554	0.075*	
C7	1.09807 (14)	0.5642 (3)	−0.01655 (9)	0.0537 (8)	
H7A	1.1060	0.4959	−0.0319	0.064*	
C8	1.07577 (12)	0.5533 (3)	0.02110 (8)	0.0406 (7)	
C9	1.05903 (14)	0.3255 (3)	0.02976 (10)	0.0501 (8)	
H9A	1.0331	0.3204	0.0070	0.060*	
H9B	1.0417	0.2747	0.0501	0.060*	
C10	1.11975 (14)	0.2738 (3)	0.01894 (9)	0.0473 (8)	
C11	1.17289 (16)	0.3269 (4)	0.02893 (11)	0.0658 (10)	
H11	1.1728	0.4014	0.0420	0.079*	
C12	1.22716 (18)	0.2714 (5)	0.01989 (13)	0.0873 (13)	
H12A	1.2630	0.3087	0.0270	0.105*	
C13	1.2278 (2)	0.1625 (4)	0.00056 (16)	0.0961 (16)	
H13A	1.2640	0.1237	−0.0047	0.115*	
C14	1.1750 (2)	0.1107 (4)	−0.01101 (15)	0.0965 (16)	
H14	1.1755	0.0380	−0.0251	0.116*	
C15	1.12055 (18)	0.1658 (3)	−0.00185 (12)	0.0714 (11)	
H15	1.0848	0.1298	−0.0097	0.086*	
C16	0.96472 (12)	0.4538 (3)	0.17165 (8)	0.0395 (7)	
H16A	0.9876	0.3936	0.1862	0.047*	
H16B	0.9282	0.4160	0.1622	0.047*	
C17	0.95091 (11)	0.5617 (3)	0.19663 (8)	0.0372 (6)	
C18	0.93908 (12)	0.7491 (3)	0.21505 (8)	0.0415 (7)	
C19	0.93622 (14)	0.8760 (3)	0.21773 (10)	0.0520 (8)	
H19	0.9469	0.9257	0.1968	0.062*	
C20	0.91687 (15)	0.9248 (4)	0.25268 (11)	0.0640 (10)	
H20	0.9142	1.0092	0.2552	0.077*	
C21	0.90137 (16)	0.8517 (4)	0.28403 (11)	0.0711 (11)	
H21	0.8887	0.8885	0.3072	0.085*	
C22	0.90411 (15)	0.7264 (4)	0.28217 (9)	0.0620 (10)	
H22	0.8939	0.6775	0.3034	0.074*	
C23	0.92308 (13)	0.6764 (3)	0.24668 (9)	0.0464 (7)	
C24	0.91835 (14)	0.4451 (3)	0.25692 (10)	0.0588 (9)	
H24A	0.9222	0.4638	0.2846	0.071*	
H24B	0.9480	0.3834	0.2505	0.071*	
C25	0.85704 (15)	0.3937 (3)	0.24952 (9)	0.0495 (8)	
C26	0.8504 (2)	0.2812 (4)	0.23218 (11)	0.0736 (11)	
H26	0.8840	0.2361	0.2251	0.088*	
C27	0.7934 (3)	0.2347 (4)	0.22523 (12)	0.0909 (15)	
H27	0.7892	0.1585	0.2135	0.109*	
C28	0.7437 (2)	0.2995 (5)	0.23540 (12)	0.0867 (13)	
H28	0.7057	0.2684	0.2304	0.104*	
C29	0.75037 (18)	0.4100 (4)	0.25290 (13)	0.0795 (12)	
H29	0.7166	0.4543	0.2602	0.095*	
C30	0.80625 (15)	0.4568 (3)	0.25995 (11)	0.0624 (10)	
H30	0.8099	0.5328	0.2720	0.075*	
C31	1.11670 (13)	0.8675 (2)	0.13666 (8)	0.0393 (7)	
C32	1.13719 (14)	0.9772 (3)	0.11795 (9)	0.0444 (7)	
C33	1.19481 (15)	1.0064 (3)	0.11096 (9)	0.0530 (8)	
H33	1.2048	1.0789	0.0983	0.064*	
C34	1.23870 (14)	0.9258 (3)	0.12305 (9)	0.0518 (8)	
C35	1.22401 (14)	0.8190 (3)	0.14234 (9)	0.0524 (8)	
H35	1.2540	0.7660	0.1507	0.063*	
C36	1.16454 (13)	0.7910 (3)	0.14919 (8)	0.0426 (7)	
C37	0.88981 (13)	0.8760 (3)	0.11454 (9)	0.0431 (7)	
C38	0.86812 (14)	0.9924 (3)	0.12730 (9)	0.0470 (7)	
C39	0.81040 (14)	1.0163 (3)	0.13732 (9)	0.0516 (8)	
H39	0.7994	1.0922	0.1473	0.062*	
C40	0.76829 (13)	0.9245 (3)	0.13228 (9)	0.0498 (8)	
C41	0.78354 (14)	0.8138 (3)	0.11696 (9)	0.0483 (8)	
H41	0.7543	0.7552	0.1121	0.058*	
C42	0.84299 (13)	0.7896 (3)	0.10876 (9)	0.0440 (7)	
Mn1	1.000034 (19)	0.71354 (4)	0.128474 (12)	0.03633 (12)	
N1	1.04023 (10)	0.61681 (19)	0.07995 (7)	0.0350 (5)	
N2	1.05970 (10)	0.4524 (2)	0.04352 (7)	0.0392 (6)	
N3	0.95682 (10)	0.6748 (2)	0.18400 (7)	0.0382 (5)	
N4	0.93111 (10)	0.5560 (2)	0.23421 (7)	0.0449 (6)	
N5	1.09112 (14)	1.0645 (3)	0.10549 (10)	0.0596 (7)	
N6	1.30101 (15)	0.9529 (4)	0.11544 (10)	0.0787 (11)	
N7	1.15152 (15)	0.6789 (3)	0.17020 (8)	0.0570 (7)	
N8	0.91170 (14)	1.0924 (3)	0.12996 (11)	0.0608 (8)	
N9	0.70641 (13)	0.9454 (3)	0.14359 (9)	0.0637 (8)	
N10	0.85563 (12)	0.6699 (2)	0.09254 (8)	0.0490 (6)	
O1	0.99855 (9)	0.50068 (17)	0.14027 (6)	0.0494 (5)	
O2	1.06126 (9)	0.84978 (19)	0.14132 (7)	0.0514 (5)	
O3	1.06283 (16)	1.0451 (3)	0.07692 (10)	0.1071 (11)	
O4	1.0964 (14)	1.1670 (8)	0.1181 (5)	0.093 (5)	0.54 (5)
O5	1.31231 (13)	1.0489 (3)	0.09751 (9)	0.0979 (11)	
O6	1.33851 (13)	0.8803 (4)	0.12623 (9)	0.1113 (13)	
O7	1.19379 (13)	0.6213 (2)	0.18350 (8)	0.0813 (8)	
O8	1.09936 (14)	0.6462 (2)	0.17412 (9)	0.0867 (9)	
O9	0.94519 (9)	0.85772 (18)	0.10919 (7)	0.0518 (5)	
O10	0.92558 (17)	1.1310 (3)	0.16102 (11)	0.1215 (13)	
O11	0.93154 (16)	1.1317 (3)	0.09981 (11)	0.1153 (12)	
O12	0.69430 (11)	1.0404 (3)	0.16102 (8)	0.0762 (8)	
O13	0.66916 (12)	0.8668 (3)	0.13503 (9)	0.0861 (9)	
O14	0.81472 (10)	0.6130 (2)	0.07709 (7)	0.0633 (6)	
O15	0.90714 (10)	0.6290 (2)	0.09460 (7)	0.0661 (7)	
O4'	1.0663 (13)	1.124 (3)	0.1326 (9)	0.108 (11)	0.46 (5)

### Atomic displacement parameters (Å^2^)

**Table:**

	*U*^11^	*U*^22^	*U*^33^	*U*^12^	*U*^13^	*U*^23^
C1	0.0403 (17)	0.0320 (14)	0.0500 (17)	0.0064 (12)	0.0027 (13)	−0.0041 (13)
C2	0.0270 (14)	0.0347 (15)	0.0437 (16)	0.0060 (11)	−0.0008 (12)	−0.0072 (12)
C3	0.0324 (15)	0.0397 (16)	0.0378 (15)	0.0011 (12)	0.0026 (12)	−0.0051 (13)
C4	0.057 (2)	0.0441 (18)	0.0478 (18)	−0.0051 (16)	0.0038 (15)	−0.0020 (15)
C5	0.067 (2)	0.059 (2)	0.059 (2)	−0.0122 (19)	0.0074 (18)	0.0096 (18)
C6	0.058 (2)	0.085 (3)	0.0446 (19)	−0.003 (2)	0.0090 (16)	0.0046 (19)
C7	0.050 (2)	0.068 (2)	0.0432 (18)	0.0085 (17)	0.0049 (15)	−0.0125 (17)
C8	0.0331 (15)	0.0480 (17)	0.0407 (16)	0.0056 (13)	0.0007 (12)	−0.0055 (14)
C9	0.0483 (19)	0.0403 (17)	0.062 (2)	0.0096 (14)	−0.0043 (16)	−0.0180 (15)
C10	0.052 (2)	0.0424 (17)	0.0471 (18)	0.0133 (15)	0.0032 (15)	−0.0046 (14)
C11	0.052 (2)	0.080 (3)	0.065 (2)	0.021 (2)	−0.0081 (18)	−0.020 (2)
C12	0.051 (2)	0.110 (4)	0.101 (3)	0.022 (2)	0.000 (2)	−0.001 (3)
C13	0.077 (3)	0.079 (3)	0.132 (4)	0.039 (3)	0.039 (3)	0.016 (3)
C14	0.105 (4)	0.046 (2)	0.138 (4)	0.021 (2)	0.059 (3)	−0.008 (2)
C15	0.074 (3)	0.0424 (19)	0.098 (3)	0.0079 (18)	0.029 (2)	−0.0154 (19)
C16	0.0313 (15)	0.0396 (16)	0.0475 (17)	−0.0050 (13)	0.0015 (13)	0.0079 (13)
C17	0.0243 (14)	0.0480 (17)	0.0394 (16)	−0.0005 (12)	0.0016 (12)	0.0021 (13)
C18	0.0289 (15)	0.0544 (19)	0.0411 (16)	−0.0018 (13)	0.0023 (12)	−0.0070 (14)
C19	0.0434 (18)	0.053 (2)	0.060 (2)	0.0032 (15)	−0.0005 (15)	−0.0146 (16)
C20	0.048 (2)	0.077 (3)	0.068 (2)	0.0103 (18)	−0.0015 (18)	−0.031 (2)
C21	0.049 (2)	0.107 (3)	0.058 (2)	0.006 (2)	0.0021 (18)	−0.035 (2)
C22	0.045 (2)	0.102 (3)	0.0389 (18)	−0.001 (2)	0.0030 (15)	−0.006 (2)
C23	0.0332 (16)	0.065 (2)	0.0408 (17)	−0.0007 (15)	0.0001 (13)	−0.0056 (15)
C24	0.046 (2)	0.077 (2)	0.053 (2)	0.0041 (18)	0.0035 (15)	0.0287 (18)
C25	0.053 (2)	0.052 (2)	0.0443 (18)	0.0009 (16)	0.0103 (15)	0.0167 (15)
C26	0.083 (3)	0.066 (3)	0.071 (3)	0.005 (2)	0.032 (2)	0.008 (2)
C27	0.130 (4)	0.072 (3)	0.071 (3)	−0.032 (3)	0.030 (3)	−0.017 (2)
C28	0.076 (3)	0.106 (4)	0.078 (3)	−0.034 (3)	0.010 (2)	−0.001 (3)
C29	0.052 (2)	0.079 (3)	0.108 (3)	−0.008 (2)	0.012 (2)	−0.002 (3)
C30	0.047 (2)	0.056 (2)	0.083 (3)	−0.0024 (17)	0.0089 (18)	0.0014 (19)
C31	0.0406 (17)	0.0337 (15)	0.0436 (17)	−0.0019 (13)	0.0001 (13)	−0.0086 (12)
C32	0.0467 (19)	0.0385 (16)	0.0480 (18)	−0.0031 (14)	−0.0001 (14)	−0.0064 (14)
C33	0.057 (2)	0.0525 (19)	0.050 (2)	−0.0179 (17)	0.0062 (16)	−0.0060 (16)
C34	0.0372 (18)	0.069 (2)	0.0490 (19)	−0.0118 (16)	0.0067 (14)	−0.0154 (17)
C35	0.0433 (18)	0.067 (2)	0.0474 (18)	0.0107 (17)	−0.0055 (15)	−0.0134 (16)
C36	0.0463 (18)	0.0404 (16)	0.0411 (16)	0.0011 (14)	0.0021 (13)	−0.0039 (14)
C37	0.0351 (17)	0.0477 (17)	0.0465 (17)	0.0084 (14)	−0.0004 (13)	0.0048 (14)
C38	0.0456 (18)	0.0411 (17)	0.0542 (19)	0.0049 (14)	−0.0028 (15)	0.0031 (15)
C39	0.053 (2)	0.0501 (19)	0.052 (2)	0.0165 (16)	0.0019 (16)	0.0025 (15)
C40	0.0365 (17)	0.061 (2)	0.0521 (19)	0.0141 (15)	0.0064 (14)	0.0064 (16)
C41	0.0388 (17)	0.0531 (19)	0.0529 (19)	0.0041 (14)	0.0030 (14)	0.0045 (15)
C42	0.0398 (17)	0.0463 (17)	0.0458 (17)	0.0059 (14)	0.0033 (13)	−0.0021 (14)
Mn1	0.0378 (2)	0.02768 (19)	0.0435 (2)	−0.00007 (19)	0.0120 (2)	−0.00267 (18)
N1	0.0309 (12)	0.0315 (12)	0.0425 (13)	0.0031 (10)	0.0044 (10)	−0.0036 (10)
N2	0.0383 (14)	0.0343 (13)	0.0450 (14)	0.0058 (11)	0.0019 (11)	−0.0103 (11)
N3	0.0325 (13)	0.0415 (13)	0.0405 (13)	−0.0025 (11)	0.0057 (10)	−0.0017 (11)
N4	0.0334 (13)	0.0618 (17)	0.0394 (14)	−0.0020 (12)	0.0041 (11)	0.0112 (12)
N5	0.065 (2)	0.0432 (16)	0.070 (2)	0.0043 (15)	−0.0056 (17)	−0.0050 (15)
N6	0.049 (2)	0.127 (3)	0.060 (2)	−0.021 (2)	0.0099 (17)	−0.028 (2)
N7	0.071 (2)	0.0478 (17)	0.0519 (17)	0.0090 (16)	−0.0017 (16)	−0.0011 (13)
N8	0.062 (2)	0.0446 (16)	0.076 (2)	0.0006 (14)	−0.0069 (18)	0.0024 (17)
N9	0.0457 (18)	0.080 (2)	0.065 (2)	0.0221 (17)	0.0109 (15)	0.0120 (18)
N10	0.0452 (16)	0.0499 (16)	0.0518 (16)	−0.0008 (13)	0.0044 (13)	−0.0078 (13)
O1	0.0606 (13)	0.0343 (10)	0.0532 (12)	−0.0033 (10)	0.0250 (11)	−0.0027 (9)
O2	0.0342 (11)	0.0486 (13)	0.0714 (15)	−0.0064 (10)	0.0051 (10)	−0.0056 (11)
O3	0.125 (3)	0.102 (2)	0.094 (2)	0.047 (2)	−0.039 (2)	−0.012 (2)
O4	0.136 (13)	0.036 (4)	0.107 (6)	0.018 (4)	−0.019 (7)	−0.011 (4)
O5	0.075 (2)	0.126 (3)	0.093 (2)	−0.050 (2)	0.0315 (17)	−0.029 (2)
O6	0.0403 (16)	0.205 (4)	0.089 (2)	0.010 (2)	−0.0017 (15)	−0.016 (2)
O7	0.105 (2)	0.0704 (17)	0.0686 (17)	0.0253 (16)	−0.0214 (16)	0.0119 (14)
O8	0.082 (2)	0.0683 (18)	0.110 (2)	−0.0113 (16)	0.0096 (17)	0.0382 (16)
O9	0.0345 (12)	0.0459 (12)	0.0750 (15)	0.0048 (10)	0.0033 (10)	0.0071 (11)
O10	0.147 (3)	0.112 (3)	0.105 (3)	−0.055 (2)	−0.009 (2)	−0.025 (2)
O11	0.126 (3)	0.103 (3)	0.117 (3)	−0.044 (2)	−0.014 (2)	0.041 (2)
O12	0.0657 (17)	0.090 (2)	0.0732 (18)	0.0367 (15)	0.0155 (14)	−0.0007 (15)
O13	0.0426 (15)	0.102 (2)	0.114 (2)	0.0076 (15)	0.0197 (15)	0.0045 (19)
O14	0.0536 (15)	0.0650 (15)	0.0713 (16)	−0.0067 (12)	−0.0018 (12)	−0.0176 (13)
O15	0.0456 (14)	0.0638 (15)	0.0888 (18)	0.0143 (12)	−0.0026 (12)	−0.0323 (14)
O4'	0.104 (11)	0.099 (15)	0.122 (13)	0.047 (11)	−0.040 (10)	−0.064 (12)

### Geometric parameters (Å, º)

**Table:**

Mn1—O2	2.069 (2)	C16—H16B	0.9700
Mn1—O9	2.102 (2)	C17—N3	1.316 (3)
Mn1—N1	2.160 (2)	C17—N4	1.358 (3)
Mn1—N3	2.168 (2)	C18—C23	1.387 (4)
Mn1—O1	2.363 (2)	C18—N3	1.392 (3)
N5—O3	1.181 (4)	C18—C19	1.393 (4)
N5—O4	1.206 (9)	C19—C20	1.376 (4)
N5—O4'	1.259 (18)	C19—H19	0.9300
N6—O6	1.211 (5)	C20—C21	1.380 (5)
N6—O5	1.241 (5)	C20—H20	0.9300
N7—O7	1.222 (3)	C21—C22	1.373 (5)
N7—O8	1.225 (4)	C21—H21	0.9300
N8—O10	1.182 (4)	C22—C23	1.395 (4)
N8—O11	1.200 (4)	C22—H22	0.9300
N9—O12	1.227 (4)	C23—N4	1.396 (4)
N9—O13	1.231 (4)	C24—N4	1.467 (4)
N10—O14	1.224 (3)	C24—C25	1.501 (4)
N10—O15	1.236 (3)	C24—H24A	0.9700
C1—O1	1.411 (3)	C24—H24B	0.9700
C1—C2	1.494 (4)	C25—C26	1.373 (5)
C1—H1A	0.9700	C25—C30	1.374 (4)
C1—H1B	0.9700	C26—C27	1.391 (6)
C2—N1	1.324 (3)	C26—H26	0.9300
C2—N2	1.350 (3)	C27—C28	1.360 (6)
C3—C4	1.388 (4)	C27—H27	0.9300
C3—C8	1.393 (4)	C28—C29	1.356 (6)
C3—N1	1.394 (3)	C28—H28	0.9300
C4—C5	1.379 (4)	C29—C30	1.370 (5)
C4—H4	0.9300	C29—H29	0.9300
C5—C6	1.391 (5)	C30—H30	0.9300
C5—H5A	0.9300	C31—O2	1.263 (3)
C6—C7	1.372 (5)	C31—C36	1.422 (4)
C6—H6	0.9300	C31—C32	1.434 (4)
C7—C8	1.383 (4)	C32—C33	1.347 (4)
C7—H7A	0.9300	C32—N5	1.467 (4)
C8—N2	1.390 (4)	C33—C34	1.381 (5)
C9—N2	1.465 (3)	C33—H33	0.9300
C9—C10	1.514 (4)	C34—C35	1.381 (5)
C9—H9A	0.9700	C34—N6	1.446 (4)
C9—H9B	0.9700	C35—C36	1.382 (4)
C10—C11	1.364 (5)	C35—H35	0.9300
C10—C15	1.378 (4)	C36—N7	1.449 (4)
C11—C12	1.389 (5)	C37—O9	1.265 (3)
C11—H11	0.9300	C37—C42	1.422 (4)
C12—C13	1.362 (6)	C37—C38	1.430 (4)
C12—H12A	0.9300	C38—C39	1.358 (4)
C13—C14	1.365 (6)	C38—N8	1.466 (4)
C13—H13A	0.9300	C39—C40	1.386 (4)
C14—C15	1.393 (5)	C39—H39	0.9300
C14—H14	0.9300	C40—C41	1.362 (4)
C15—H15	0.9300	C40—N9	1.452 (4)
C16—O1	1.407 (3)	C41—C42	1.382 (4)
C16—C17	1.488 (4)	C41—H41	0.9300
C16—H16A	0.9700	C42—N10	1.450 (4)
			
O1—C1—C2	105.4 (2)	C29—C28—C27	119.2 (4)
O1—C1—H1A	110.7	C29—C28—H28	120.4
C2—C1—H1A	110.7	C27—C28—H28	120.4
O1—C1—H1B	110.7	C28—C29—C30	120.7 (4)
C2—C1—H1B	110.7	C28—C29—H29	119.7
H1A—C1—H1B	108.8	C30—C29—H29	119.7
N1—C2—N2	113.0 (3)	C29—C30—C25	121.2 (4)
N1—C2—C1	122.5 (2)	C29—C30—H30	119.4
N2—C2—C1	124.6 (2)	C25—C30—H30	119.4
C4—C3—C8	121.1 (3)	O2—C31—C36	127.4 (3)
C4—C3—N1	129.9 (3)	O2—C31—C32	119.8 (3)
C8—C3—N1	109.0 (2)	C36—C31—C32	112.7 (3)
C5—C4—C3	116.9 (3)	C33—C32—C31	125.6 (3)
C5—C4—H4	121.6	C33—C32—N5	117.6 (3)
C3—C4—H4	121.6	C31—C32—N5	116.8 (3)
C4—C5—C6	121.6 (3)	C32—C33—C34	118.2 (3)
C4—C5—H5A	119.2	C32—C33—H33	120.9
C6—C5—H5A	119.2	C34—C33—H33	120.9
C7—C6—C5	121.8 (3)	C35—C34—C33	120.9 (3)
C7—C6—H6	119.1	C35—C34—N6	119.2 (4)
C5—C6—H6	119.1	C33—C34—N6	119.9 (4)
C6—C7—C8	116.9 (3)	C34—C35—C36	119.7 (3)
C6—C7—H7A	121.6	C34—C35—H35	120.1
C8—C7—H7A	121.6	C36—C35—H35	120.1
C7—C8—N2	132.3 (3)	C35—C36—C31	122.7 (3)
C7—C8—C3	121.7 (3)	C35—C36—N7	117.6 (3)
N2—C8—C3	106.0 (2)	C31—C36—N7	119.7 (3)
N2—C9—C10	115.0 (3)	O9—C37—C42	126.4 (3)
N2—C9—H9A	108.5	O9—C37—C38	121.0 (3)
C10—C9—H9A	108.5	C42—C37—C38	112.6 (3)
N2—C9—H9B	108.5	C39—C38—C37	124.7 (3)
C10—C9—H9B	108.5	C39—C38—N8	118.0 (3)
H9A—C9—H9B	107.5	C37—C38—N8	117.3 (3)
C11—C10—C15	118.8 (3)	C38—C39—C40	118.2 (3)
C11—C10—C9	124.0 (3)	C38—C39—H39	120.9
C15—C10—C9	117.2 (3)	C40—C39—H39	120.9
C10—C11—C12	121.2 (4)	C41—C40—C39	121.4 (3)
C10—C11—H11	119.4	C41—C40—N9	118.7 (3)
C12—C11—H11	119.4	C39—C40—N9	119.9 (3)
C13—C12—C11	119.9 (4)	C40—C41—C42	119.2 (3)
C13—C12—H12A	120.1	C40—C41—H41	120.4
C11—C12—H12A	120.1	C42—C41—H41	120.4
C12—C13—C14	119.6 (4)	C41—C42—C37	123.4 (3)
C12—C13—H13A	120.2	C41—C42—N10	115.9 (3)
C14—C13—H13A	120.2	C37—C42—N10	120.6 (3)
C13—C14—C15	120.6 (4)	O2—Mn1—O9	84.88 (9)
C13—C14—H14	119.7	O2—Mn1—N1	103.93 (8)
C15—C14—H14	119.7	O9—Mn1—N1	111.67 (9)
C10—C15—C14	119.8 (4)	O2—Mn1—N3	104.44 (9)
C10—C15—H15	120.1	O9—Mn1—N3	99.28 (9)
C14—C15—H15	120.1	N1—Mn1—N3	139.39 (9)
O1—C16—C17	105.0 (2)	O2—Mn1—O1	133.06 (8)
O1—C16—H16A	110.8	O9—Mn1—O1	141.66 (8)
C17—C16—H16A	110.8	N1—Mn1—O1	69.75 (8)
O1—C16—H16B	110.8	N3—Mn1—O1	69.66 (8)
C17—C16—H16B	110.8	C2—N1—C3	105.4 (2)
H16A—C16—H16B	108.8	C2—N1—Mn1	121.20 (19)
N3—C17—N4	112.7 (3)	C3—N1—Mn1	132.84 (18)
N3—C17—C16	122.5 (2)	C2—N2—C8	106.7 (2)
N4—C17—C16	124.9 (3)	C2—N2—C9	127.5 (3)
C23—C18—N3	109.4 (3)	C8—N2—C9	125.3 (2)
C23—C18—C19	120.5 (3)	C17—N3—C18	105.7 (2)
N3—C18—C19	130.1 (3)	C17—N3—Mn1	120.95 (18)
C20—C19—C18	117.2 (3)	C18—N3—Mn1	132.66 (19)
C20—C19—H19	121.4	C17—N4—C23	106.6 (2)
C18—C19—H19	121.4	C17—N4—C24	126.9 (3)
C19—C20—C21	121.8 (4)	C23—N4—C24	126.4 (3)
C19—C20—H20	119.1	O3—N5—O4	120.8 (6)
C21—C20—H20	119.1	O3—N5—O4'	117.6 (6)
C22—C21—C20	122.0 (3)	O4—N5—O4'	45.7 (9)
C22—C21—H21	119.0	O3—N5—C32	119.8 (3)
C20—C21—H21	119.0	O4—N5—C32	115.7 (10)
C21—C22—C23	116.4 (4)	O4'—N5—C32	115.6 (12)
C21—C22—H22	121.8	O6—N6—O5	124.3 (4)
C23—C22—H22	121.8	O6—N6—C34	118.4 (4)
C18—C23—C22	122.0 (3)	O5—N6—C34	117.2 (4)
C18—C23—N4	105.7 (3)	O7—N7—O8	122.9 (3)
C22—C23—N4	132.4 (3)	O7—N7—C36	117.7 (3)
N4—C24—C25	113.4 (3)	O8—N7—C36	119.4 (3)
N4—C24—H24A	108.9	O10—N8—O11	123.0 (4)
C25—C24—H24A	108.9	O10—N8—C38	119.7 (3)
N4—C24—H24B	108.9	O11—N8—C38	117.3 (3)
C25—C24—H24B	108.9	O12—N9—O13	123.9 (3)
H24A—C24—H24B	107.7	O12—N9—C40	118.1 (3)
C26—C25—C30	118.2 (3)	O13—N9—C40	118.0 (3)
C26—C25—C24	120.4 (3)	O14—N10—O15	122.3 (3)
C30—C25—C24	121.4 (3)	O14—N10—C42	118.6 (3)
C25—C26—C27	120.0 (4)	O15—N10—C42	119.1 (3)
C25—C26—H26	120.0	C16—O1—C1	116.8 (2)
C27—C26—H26	120.0	C16—O1—Mn1	119.74 (16)
C28—C27—C26	120.8 (4)	C1—O1—Mn1	119.88 (16)
C28—C27—H27	119.6	C31—O2—Mn1	136.98 (19)
C26—C27—H27	119.6	C37—O9—Mn1	129.94 (19)
			
O1—C1—C2—N1	−4.2 (4)	O2—Mn1—N1—C3	−50.9 (3)
O1—C1—C2—N2	177.5 (2)	O9—Mn1—N1—C3	39.1 (3)
C8—C3—C4—C5	0.2 (5)	N3—Mn1—N1—C3	176.0 (2)
N1—C3—C4—C5	−177.4 (3)	O1—Mn1—N1—C3	178.0 (3)
C3—C4—C5—C6	0.0 (5)	N1—C2—N2—C8	−1.6 (3)
C4—C5—C6—C7	−0.4 (6)	C1—C2—N2—C8	176.9 (3)
C5—C6—C7—C8	0.5 (5)	N1—C2—N2—C9	−173.8 (3)
C6—C7—C8—N2	178.5 (3)	C1—C2—N2—C9	4.7 (4)
C6—C7—C8—C3	−0.3 (5)	C7—C8—N2—C2	−177.4 (3)
C4—C3—C8—C7	0.0 (4)	C3—C8—N2—C2	1.6 (3)
N1—C3—C8—C7	178.0 (3)	C7—C8—N2—C9	−4.9 (5)
C4—C3—C8—N2	−179.1 (3)	C3—C8—N2—C9	174.0 (3)
N1—C3—C8—N2	−1.1 (3)	C10—C9—N2—C2	−124.2 (3)
N2—C9—C10—C11	14.4 (5)	C10—C9—N2—C8	64.9 (4)
N2—C9—C10—C15	−166.4 (3)	N4—C17—N3—C18	−0.8 (3)
C15—C10—C11—C12	−2.6 (6)	C16—C17—N3—C18	179.1 (2)
C9—C10—C11—C12	176.6 (4)	N4—C17—N3—Mn1	170.76 (17)
C10—C11—C12—C13	0.3 (7)	C16—C17—N3—Mn1	−9.3 (4)
C11—C12—C13—C14	2.3 (7)	C23—C18—N3—C17	0.7 (3)
C12—C13—C14—C15	−2.6 (8)	C19—C18—N3—C17	−179.7 (3)
C11—C10—C15—C14	2.3 (6)	C23—C18—N3—Mn1	−169.4 (2)
C9—C10—C15—C14	−176.9 (4)	C19—C18—N3—Mn1	10.1 (5)
C13—C14—C15—C10	0.3 (7)	O2—Mn1—N3—C17	−131.0 (2)
O1—C16—C17—N3	15.9 (4)	O9—Mn1—N3—C17	141.9 (2)
O1—C16—C17—N4	−164.2 (2)	N1—Mn1—N3—C17	2.0 (3)
C23—C18—C19—C20	−0.2 (5)	O1—Mn1—N3—C17	0.0 (2)
N3—C18—C19—C20	−179.8 (3)	O2—Mn1—N3—C18	37.9 (3)
C18—C19—C20—C21	0.6 (5)	O9—Mn1—N3—C18	−49.2 (3)
C19—C20—C21—C22	−0.3 (6)	N1—Mn1—N3—C18	170.9 (2)
C20—C21—C22—C23	−0.4 (5)	O1—Mn1—N3—C18	168.9 (3)
N3—C18—C23—C22	179.2 (3)	N3—C17—N4—C23	0.6 (3)
C19—C18—C23—C22	−0.5 (5)	C16—C17—N4—C23	−179.3 (3)
N3—C18—C23—N4	−0.3 (3)	N3—C17—N4—C24	178.4 (3)
C19—C18—C23—N4	−180.0 (3)	C16—C17—N4—C24	−1.5 (4)
C21—C22—C23—C18	0.8 (5)	C18—C23—N4—C17	−0.1 (3)
C21—C22—C23—N4	−179.9 (3)	C22—C23—N4—C17	−179.5 (3)
N4—C24—C25—C26	114.8 (4)	C18—C23—N4—C24	−178.0 (3)
N4—C24—C25—C30	−65.5 (4)	C22—C23—N4—C24	2.6 (5)
C30—C25—C26—C27	0.7 (5)	C25—C24—N4—C17	−83.4 (4)
C24—C25—C26—C27	−179.6 (3)	C25—C24—N4—C23	94.0 (4)
C25—C26—C27—C28	0.1 (6)	C33—C32—N5—O3	103.3 (4)
C26—C27—C28—C29	−0.8 (7)	C31—C32—N5—O3	−76.8 (4)
C27—C28—C29—C30	0.7 (7)	C33—C32—N5—O4	−55.5 (16)
C28—C29—C30—C25	0.0 (6)	C31—C32—N5—O4	124.4 (16)
C26—C25—C30—C29	−0.7 (5)	C33—C32—N5—O4'	−107 (2)
C24—C25—C30—C29	179.6 (3)	C31—C32—N5—O4'	73 (2)
O2—C31—C32—C33	−179.4 (3)	C35—C34—N6—O6	0.2 (5)
C36—C31—C32—C33	2.5 (4)	C33—C34—N6—O6	−179.7 (3)
O2—C31—C32—N5	0.6 (4)	C35—C34—N6—O5	178.9 (3)
C36—C31—C32—N5	−177.5 (3)	C33—C34—N6—O5	−1.0 (5)
C31—C32—C33—C34	−0.9 (5)	C35—C36—N7—O7	6.1 (4)
N5—C32—C33—C34	179.1 (3)	C31—C36—N7—O7	−173.6 (3)
C32—C33—C34—C35	−1.0 (5)	C35—C36—N7—O8	−174.3 (3)
C32—C33—C34—N6	178.9 (3)	C31—C36—N7—O8	6.0 (4)
C33—C34—C35—C36	1.0 (5)	C39—C38—N8—O10	68.6 (5)
N6—C34—C35—C36	−178.9 (3)	C37—C38—N8—O10	−110.9 (4)
C34—C35—C36—C31	0.9 (5)	C39—C38—N8—O11	−111.0 (4)
C34—C35—C36—N7	−178.9 (3)	C37—C38—N8—O11	69.4 (4)
O2—C31—C36—C35	179.7 (3)	C41—C40—N9—O12	172.6 (3)
C32—C31—C36—C35	−2.4 (4)	C39—C40—N9—O12	−7.1 (5)
O2—C31—C36—N7	−0.6 (4)	C41—C40—N9—O13	−7.5 (5)
C32—C31—C36—N7	177.3 (3)	C39—C40—N9—O13	172.7 (3)
O9—C37—C38—C39	−173.4 (3)	C41—C42—N10—O14	20.3 (4)
C42—C37—C38—C39	7.2 (5)	C37—C42—N10—O14	−158.2 (3)
O9—C37—C38—N8	6.1 (4)	C41—C42—N10—O15	−160.1 (3)
C42—C37—C38—N8	−173.3 (3)	C37—C42—N10—O15	21.5 (4)
C37—C38—C39—C40	−4.9 (5)	C17—C16—O1—C1	−174.1 (2)
N8—C38—C39—C40	175.6 (3)	C17—C16—O1—Mn1	−15.3 (3)
C38—C39—C40—C41	−1.3 (5)	C2—C1—O1—C16	169.8 (2)
C38—C39—C40—N9	178.4 (3)	C2—C1—O1—Mn1	11.0 (3)
C39—C40—C41—C42	4.4 (5)	O2—Mn1—O1—C16	100.6 (2)
N9—C40—C41—C42	−175.3 (3)	O9—Mn1—O1—C16	−69.3 (2)
C40—C41—C42—C37	−1.6 (5)	N1—Mn1—O1—C16	−169.0 (2)
C40—C41—C42—N10	180.0 (3)	N3—Mn1—O1—C16	9.61 (19)
O9—C37—C42—C41	176.8 (3)	O2—Mn1—O1—C1	−101.3 (2)
C38—C37—C42—C41	−3.8 (4)	O9—Mn1—O1—C1	88.8 (2)
O9—C37—C42—N10	−4.9 (5)	N1—Mn1—O1—C1	−10.92 (19)
C38—C37—C42—N10	174.5 (3)	N3—Mn1—O1—C1	167.7 (2)
N2—C2—N1—C3	0.9 (3)	C36—C31—O2—Mn1	−55.3 (5)
C1—C2—N1—C3	−177.6 (2)	C32—C31—O2—Mn1	126.9 (3)
N2—C2—N1—Mn1	173.22 (17)	O9—Mn1—O2—C31	−135.1 (3)
C1—C2—N1—Mn1	−5.3 (4)	N1—Mn1—O2—C31	−24.0 (3)
C4—C3—N1—C2	178.0 (3)	N3—Mn1—O2—C31	126.6 (3)
C8—C3—N1—C2	0.2 (3)	O1—Mn1—O2—C31	51.1 (3)
C4—C3—N1—Mn1	7.0 (5)	C42—C37—O9—Mn1	−48.7 (4)
C8—C3—N1—Mn1	−170.86 (19)	C38—C37—O9—Mn1	131.9 (3)
O2—Mn1—N1—C2	139.3 (2)	O2—Mn1—O9—C37	−137.0 (3)
O9—Mn1—N1—C2	−130.8 (2)	N1—Mn1—O9—C37	120.0 (3)
N3—Mn1—N1—C2	6.1 (3)	N3—Mn1—O9—C37	−33.2 (3)
O1—Mn1—N1—C2	8.1 (2)	O1—Mn1—O9—C37	35.6 (3)
